# Comparative study of the polyphenol content-related anti-inflammatory and antioxidant activities of two *Urera aurantiaca* specimens from different geographical areas

**DOI:** 10.1186/s13020-018-0181-1

**Published:** 2018-04-19

**Authors:** Carla Marrassini, Ignacio Peralta, Claudia Anesini

**Affiliations:** Facultad de Farmacia y Bioquímica, Instituto de la Química y Metabolismo del Fármaco (IQUIMEFA), Consejo Nacional de Investigaciones Científicas y Técnicas, Universidad de Buenos Aires, Buenos Aires, Argentina

**Keywords:** *Urera aurantiaca*, Inflammation, Antioxidant activity, Macrophages, Polyphenols

## Abstract

**Background:**

*Urera aurantiaca* is an Argentinean species that has been traditionally used to treat symptoms of inflammation. The aim of this study was to determine and compare the anti-inflammatory and antioxidant effects of two specimens of *Urera aurantiaca* obtained in the provinces of Salta and Misiones, which are two different geographical areas of Argentina.

**Methods:**

The anti-inflammatory activity of the extracts was tested in LPS-stimulated macrophages through the DPPH radical scavenging activity, the SOD-like activity, the reducing power and the inhibition of lipid peroxidation. The anti-inflammatory activity was also evaluated by the inhibition of albumin denaturation and proteinase inhibitory action tests. The total polyphenols, flavonoids and tannins content were quantified.

**Results:**

Both extracts were able to reduce the augmented NO release in LPS-activated macrophages and showed antioxidant and in vitro anti-inflammatory activities. The polyphenols content was higher in the extract obtained from the specimen from Salta than in that obtained in Misiones. This finding accounts for the higher anti-inflammatory and antioxidant properties obtained with the former.

**Conclusion:**

The differences in chemical composition and the biological activities observed between the extracts are probably related to the different environmental conditions found in both provinces.

**Electronic supplementary material:**

The online version of this article (10.1186/s13020-018-0181-1) contains supplementary material, which is available to authorized users.

## Background

Inflammation is a defence mechanism against tissue damage caused by either mechanical, chemical, or microbial stimuli. The main cells involved in the inflammatory response are monocytes/macrophages, polymorphonuclear leucocytes, and endothelial cells. When these cells become activated, they aggregate and infiltrate tissues where the respiratory burst is triggered to increase the oxygen consumption and the production of cytokines and reactive oxygen species (ROS), as well as other inflammation mediators [1]. The ROS that are produced during the inflammatory response facilitating it; however, when produced for prolonged periods, these mediators can promote oxidative stress and chronic inflammation-associated disorders. It is known that inflammation and oxidative stress are interrelated processes [2].

In living organisms, the energy production is achieved through oxidation processes. Thus, the aerobic metabolism entails the generation of different ROS, such as singlet oxygen, superoxide, peroxyl and hydroxyl radicals, and reactive nitrogen species (RNS), including peroxynitrite and nitric oxide (NO). Apart from their physiological activity, ROS and RNS oxidise membrane lipids, proteins and DNA, leading to diseases, such as cancer, cardiovascular diseases, cataracts, asthma, hepatitis, liver injury and immunodeficiency diseases [3].

During inflammation, ROS and RNS are largely produced by macrophages, which play a central role in both innate and adaptive immunity. Macrophages are known to perform several functions such as killing foreign organisms, presenting antigens, phagocytising foreign invaders, and producing chemical mediators, such as cytokines and those of oxidative stress. These cells interact with T and B cells, natural killer and dendritic cells, neutrophils, and fibroblasts.

The lipopolysaccharide (LPS), which is a component of cell walls and the major virulence factor of Gram-negative bacteria, can induce oxidative stress in macrophages [4].

Nowadays, there is an increasing interest in the use of plant antioxidants for dietary, pharmaceutical and cosmetic purposes. This is mainly due to their strong biological activity, exceeding those of many carcinogenic synthetic antioxidants. Many plants contain antioxidant compounds and these compounds protect cells against the damaging effects of ROS. Epidemiological studies have indicated the beneficial effects of plant antioxidants in patients with chronic diseases [5]. Thus, the antioxidant activity of plant-derived compounds is currently being studied, as regards the free radical quenching ability, mainly of polyphenol compounds.

*Urera aurantiaca* Wedd. (Urticaceae), commonly known as “ortiga colorada”, “pino guasú”, and “pica pica”, is an Argentinean native herb also growing in Paraguay, Uruguay, Bolivia, and Brazil. *U. aurantiaca* is used in folk medicine to treat rheumatic pain, varicose veins, furuncles, bruises, inflammation, tooth pain, dermal diseases, and trauma [6].

In a previous work, the in vivo anti-inflammatory and antinociceptive effects of a methanolic extract of *U. aurantiaca* from Paraguay have been reported [7]. Its modulatory effect on immune and tumoral cells during inflammation has also been demonstrated [8].

Species of the Urticaceae family have been used in traditional Chinese medicine. For example, *Laportea bulbifera* (Siebold & Zucc.) Wedd. (Urticaceae) is used for the treatment and management of urinary stones [9]; *Gonostegia hirta* (Blume) Miq. (Urticaceae) is used to treat many types of injuries [10]; *Pilea microphylla* (L.) Liebm. (Urticaceae) is used for the treatment of osteoarthritis [11]; *Pellionia repens* (Lour.) Merr. (Urticaceae) is used for the treatment of skin injuries [12]; *Boehmeria nivea* (L.) Gaudich. (Urticaceae) is employed as a pro-coagulant, to reduce swelling and as detoxifying [13].

*Urera aurantiaca* is distributed in different phytogeographic zones in Argentina. It is known that specimens of the same species growing under different environmental conditions differ in the production levels of primary and secondary metabolites. This phenomenon is due to the different metabolic requirements to accomplish long term acclimation or local adaptation, seasonal differences related to phenology or environmental changes of both biotic and abiotic factors, geographical differences involving different populations (genetic differences within a plant species), etc. [14, 15].

Considering the popular medicinal use of *U. aurantiaca* and the biological activities previously reported for this species, the objectives of this work were to analyse and compare the antioxidant effect of two species of *U. aurantiaca* obtained in the provinces of Salta and Misiones, which are two different geographical areas of Argentina. The free radicals scavenging capacity, the reducing power, the SOD-like activity, and the ability to diminish the production of NO in lipopolysaccharide (LPS)-stimulated macrophages were studied. The in vitro anti-inflammatory activity in relation to ROS generation was also studied. Finally, polyphenols such as flavonoids and tannins were quantified to relate their presence with the activities mentioned.

## Methods

The minimum standards of reporting checklist contains details of the experimental design, and resources used in this study (Additional file 1).

### Plant material

*Urera aurantiaca* Wedd. (Urticaceae) obtained in the province of Salta was collected in Orán, Salta, Argentina (23°07′01.0″S, 64°18′40.2″W) in April 2013 by P. Mercado. A voucher specimen (no. 212) was deposited at Herbario LIL Fundación Miguel Lillo, San Miguel de Tucumán, Argentina.

*Urera aurantiaca* Wedd. (Urticaceae) obtained in the province of Misiones province was collected in Eldorado, Misiones, Argentina (26°24′23.4″S, 54°41′37.3″W) in April 2013 by H.A. Keller. A voucher specimen (no. 11330) was deposited at Herbario del Instituto de Botánica del Nordeste (CTES), Corrientes, Argentina.

### Extraction

The dried aerial parts of the specimens obtained Salta and Misiones were ground to a fine powder and extracted sequentially by maceration with solvents of different polarity. Briefly, the plant powder was first extracted with dichloromethane, then with ethyl acetate, and finally, the marc was extracted with methanol. Solvents were put in contact with the plant material overnight in order to obtain an extract enriched in polyphenols. After extraction, the methanol extracts were lyophilised. Yields were 4.4% for the Salta extract and 4.6% for the Misiones extract.

### Phytochemical analysis

#### Total phenol content

The total phenol content in the extracts was determined by the Folin–Ciocalteu’s colorimetric method, as described by Singleton et al. [16]. The absorbance was measured at 760 nm and compared with a gallic acid calibration curve. Results were expressed as mg of gallic acid equivalents per gram of extract (GAE/g).

#### Flavonoids determination

The total flavonoids content was determined in the extracts by the aluminum chloride colorimetric method, as described by Chang et al. [17]. Briefly, the diluted standard solutions or the extract (0.5 ml) were mixed with 1.5 ml 95% ethanol, 0.1 ml 10% aluminum chloride, 0.1 ml 1 M potassium acetate and 2.8 ml distilled water. After incubation at room temperature for 30 min, the absorbance was measured at 415 nm. Dilutions of quercetin in ethanol were used to generate the calibration curve (125, 250, 500 and 1000 μg/ml).

#### Tannins determination

The tannin content was calculated as the difference between the contents of total phenols and the non-complex residual phenols [18] after the removal of tannins from the medium by complexation with casein, as described by Amorim et al. [19] with slight modifications. Briefly, 500 mg of casein were transferred into a 25 ml Erlenmeyer flask. Five ml of the extract and 5 ml of distilled water were then added. After 2 h, which is the time period required for the complexing of the tannins to the protein, the extract was filtered into a 10 ml volumetric flask and the volume was then adjusted to 10 ml with distilled water. The tannin content was expressed as the percentage of the total phenols that were complexed with casein.

#### High performance liquid chromatography

The HPLC method was developed and validated according to Filip et al. [20] and performed in a Varian 9000 instrument equipped with a diode array detector and a Rheodyne injector fitted with a 20 μl loop. A C18 column (Kinetex 5 μm, 150 mm × 4.6 mm) was employed. As mobile phase, solvent A: H_2_O/AcOH (98:2) and solvent B: MeOH/AcOH (98:2) were used. The elution gradient was: from 15 to 40% B in 30 min; from 40 to 75% B in 10 min; from 75 to 85% B in 5 min. The elution was performed at flow rate of 1.2 ml/min. Detection was done at 325 nm.

### Cell suspensions and culture conditions

The American Type Culture Collection (ATCC) RAW264.7 murine macrophage cell line was cultured in corlorless Dulbecco’s Modified Eagle’s medium supplemented with 10% FBS, 2 mM glutamine and 100 IU/ml penicillin and 100 μg/ml streptomycin and kept in a humidified incubator at 5% CO_2_ and 37 °C.

### Cell proliferation and nitric oxide determination

To determine cell proliferation rate and NO production, macrophages were incubated for 24 h without any treatment (basal conditions) or with LPS (1 μg/ml) alone or in the presence of different concentrations of the extracts. After incubation, the supernatant was separated to determine the NO levels and the cells were used to determine proliferation. The nitrite accumulated in the culture medium, which is an indicator of NO production, was measured by the Griess’ reaction [21]. Briefly, 100 μl of each supernatant were mixed with 50 μl of a 1% sulfanilamide solution in 5% phosphoric acid and 50 μl of a 0.1% naphthylene diamine dihydrochloride solution. The mixture was incubated at room temperature for 20 min, and the absorbance was read at 540 nm. As standard curve, serial dilutions of a NaNO_2_ solution were employed.

Results were expressed as mean ± SEM of three determinations performed in triplicate.

Cell proliferation was determined by the reduction of 3-(4,5-dimethylthiazol-2-yl)-2,5-diphenyltetrazolium bromide (MTT) (Sigma). Briefly, cells were incubated for 4 h in 100 μl RPMI 1640 and 10 μl of 5 mg/ml MTT (Sigma, St. Louis, MO, USA). After incubation, the formazan formed was dissolved in acidified isopropanol (0.04 N HCl in isopropanol) and read at 540 nm.

### 2,2-Diphenyl-1-picrylhydrazyl (DPPH) radical scavenging activity

The scavenging activity of the extracts on the stable free radical DPPH was assayed by the modified method described by Blois [22]. The bleaching rate of DPPH is monitored at a characteristic wavelength in the presence of the sample. Briefly, a volume of 0.1 ml of an aqueous dilution of the extract was mixed with 0.5 ml of a 500 μM DPPH solution in absolute ethanol and 0.4 ml of a 0.1 M Tris–HCl buffer, pH 7.4. The mixture was kept for 20 min in the darkness, and then the absorbance was read at 517 nm. The percentage of decrease of DPPH absorbance was calculated by measuring the absorbance of the sample and applying the following equation:

$$\% \;{\text{of inhibition}} = \left[ { 1- \left( {{\text{As}}/{\text{A}}0} \right)} \right] \times 100,$$

where As is absorbance of sample and A0 is the absorbance of the DPPH solution.

### Superoxide dismutase (SOD)-like activity

To determine the SOD activity, the inhibitory capacity of the extracts on the adrenaline autoxidation was evaluated. Briefly, 50 μl of the extract were treated with 910 μl of sodium phosphate buffer (0.05 M) pH: 10.7 and 1 mM adrenaline. Under these conditions, adrenaline rapidly undergoes autoxidation to produce the adrenochrome, which is a pink coloured product that can be measured at 480 nm using a UV/visible spectrophotometer in kinetic mode. The antioxidant activity of sample was evaluated as the % of epinephrine autoxidation inhibition. The absorbance was monitored at 480 nm, and the Δ absorbance/min was determined to calculate the % of inhibition as: [(Abs/min control − Abs/min sample)/Abs/min control] × 100 [23].

### Reducing power assay

The reducing power was determined according to Ferreira et al. [24], with slight modifications. Different concentrations of the extract (2.5 ml) were mixed with 2.5 ml of 200 mM sodium phosphate buffer (pH 6.6) and 2.5 ml of 1% potassium ferricyanide. The mixture was incubated at 50 °C for 20 min. After adding 2.5 ml of 10% trichloroacetic acid (w/v), the mixture was centrifuged at 3500 rpm for 10 min. The upper layer (2.5 ml) was mixed with 2.5 ml deionised water and 1 ml of 0.1% ferric chloride, and the absorbance was measured at 700 nm. The reducing power is directly proportional to the mixture absorbance.

### Inhibition of lipid peroxidation of egg yolk

The lipid peroxidation assay was done according to Dissanayake et al. [25], with minor modifications. The egg yolk was separated from the albumen and the yolk membrane was removed. A 10% v/v yolk solution was prepared in 1.15% of KCl. The solution was homogenized for 30 s and ultra sonicated for 5 min.

A 1% (w/v) of thiobarbituric acid solution was then prepared in 1.1% (w/v) SDS.

The reaction mixture contained 25 µl of the extract, 125 µl of egg yolk solution and 100 µl of distilled water. Distilled water (125 µl) was used as the control. Acetic acid (20%, 375 µl) and 1% thiobarbituric acid (375 µl) were then added. These mixtures were vortexed for 5 s and kept in a water bath 95 °C for 60 min. Butanol (1.25 ml) was added to each tube and vortexed for 5 s. After centrifuging at 3000*g* for 10 min, the butanol layer was separated. Absorbance values were measured at 532 nm.

### Inhibition of albumin denaturation

The method was performed according to Kumari et al. [26] and Sakat et al. [27], with minor modifications. The reaction mixture (0.5 ml) consisted of 0.40 ml bovine serum albumin (1% aqueous solution), 0.05 ml of the extract at different concentrations (1000, 800, 600, and 400 μg/ml), and 0.05 ml of salicylic acid (inductor of protein denaturation). As positive control, distilled water (0.05 ml) was used instead of the extracts while as negative control, no salicylic acid was employed. Samples were incubated at 37 °C for 20 min and then heated at 57 °C for 20 min. After cooling the samples, 2.5 ml phosphate buffer saline (pH 6.3) were added to each tube. Turbidity was measured spectrophotometrically at 660 nm. The percentage inhibition of protein denaturation was calculated as follows:

$${\text{Percent inhibition }} = \frac{{{\text{Abs}}_{\text{control }} {-}{\text{ Abs}}_{\text{treated}} }}{{{\text{Abs}}_{\text{Control}} }} \times 100$$

where Abs_control_ represents 100% protein denaturation.

### Proteinase inhibitory action

The antitryptic activity of the extract was assayed as described by Oyedapo and Famurewa [28], with minor modifications. The reaction mixture (1.0 ml) contained 0.03 ml trypsin (0.3 μg), 0.5 ml of the extract at different concentrations (10, 25, 50, and 100 μg/ml) and 0.5 ml of 20 mM Tris–HCl buffer (pH 7.4). The mixture was incubated at 37 °C for 5 min and then, 0.5 ml of 0.8% (w/v) casein was added. The mixture was further incubated for 20 min and 1 ml of 70% perchloric acid was added to terminate the reaction. The cloudy suspension was clarified by centrifugation and the absorbance of the supernatant was read at 280 nm against buffer as blank.

### Statistical analysis

Data were expressed as the average of triplicate values of three independent experiments. To compare two values, the Student’s *t* test was used. Multiple comparisons were performed by analysis of variance (ANOVA) and the Dunnett’s test. A p < 0.05 was considered statistically significant.

## Results

**Fig. 1:**
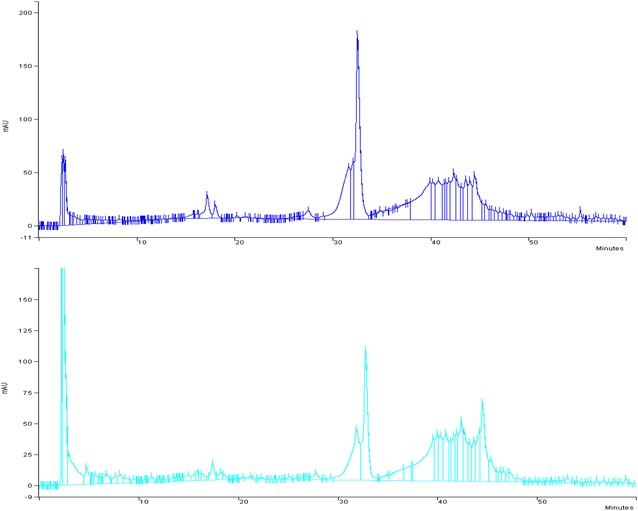
HPLC profiles: **a** Salta extract, **b** Misiones extract

Firstly, the extracts were obtained and their chemical composition was determined. The objective of the extraction was to obtain polyphenolic compounds. Once the extracts were prepared, the total polyphenols, flavonoids, and tannins were quantified. Table 1 shows that the Salta extract rendered the highest total phenolic and flavonoids content, meanwhile the Misiones extract presented a higher amount of tannins than the Salta extract. Furthermore, a chromatographic profile was also obtained by HPLC analysis. Figure 1 shows that the differences in the extracts appeared to be quantitative and not qualitative.

**Table 1:** Quantitative determination of polyphenols, flavonoids, and tannins in Salta and Misiones extracts

*Urera aurantiaca* extracts	Total polyphenols (GAE/g)	Flavonoids (%)	Tannins (%)
Salta	30.90 ± 1.8^a^	2.10 ± 0.015^a^	39.7 ± 0.2^a^
Misiones	14.05 ± 2.60^b^	1.49 ± 0.052^b^	48.2 ± 1.1^b^

Statistical differences were determined by *t*-Student test (a ≠ b: p < 0.05)

**Fig. 2:**
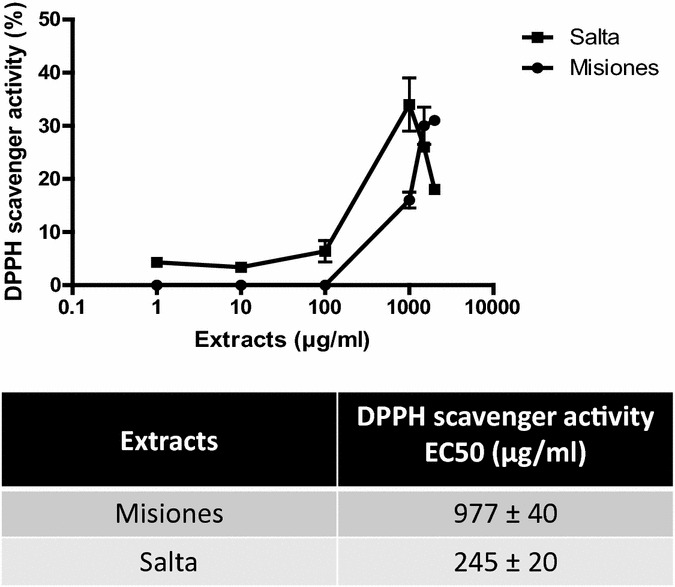
DPPH scavenger activity. **a** DPPH antioxidant activity as a function of the extract concentration. **b**–**d** Comparison between the DPPH activity and the polyphenols, flavonoids and tannins content and EC_50_ for Salta and Misiones extracts. Results represent mean ± SEM of three experiments performed in triplicate. ***p < 0.001 significant differences between extracts EC_50_

Secondly, the antioxidant activity of the extracts was measured with the DPPH test. Both extracts presented a polyphenol concentration-dependent antioxidant activity (Fig. 2a). The EC_50_ obtained with the Salta extract was fourfold lower than that of Misiones extract (Fig. 2b–d).

**Fig. 3:**
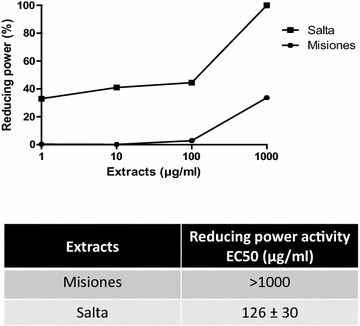
Reducing power determination. **a** Reducing power as a function of the extract concentration. **b**–**d** Comparison between the reducing power and the polyphenols, flavonoids and tannins content and EC_50_ for Salta and Misiones extracts. Results represent mean ± SEM of three experiments performed in triplicate. Significant differences between extracts EC_50_ values were determined by the *t*-Student test (***p < 0.001)

Furthermore, the reducing power of the extracts was studied. A concentration-dependent reducing power was demonstrated in both extracts (Fig. 3a). However, the Salta extract reached an inhibition of 100% at 1000 μg/ml, meanwhile for the Misiones extract, the maximum inhibition achieved was 33.7% at the same concentration. In addition, the Salta extract presented a lower EC_50_ value. In Fig. 3b–d the EC_50_ values for this activity in comparison with the polyphenols, flavonoids and tannins content is shown.

**Fig. 4:**
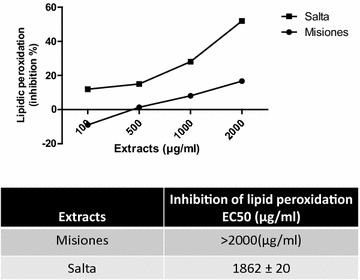
Lipid peroxidation. **a** Percentage of inhibition of lipid peroxidation as a function of the extract concentration. **b**–**d** Comparison between the lipid peroxidation inhibitory activity and the polyphenols, flavonoids and tannins content and EC_50_ for Salta and Misiones extracts. Results represent mean ± SEM of three experiments performed in triplicate. Significant differences between extracts EC_50_ were determined by the *t*-Student test (**p < 0.01)

The SOD-like activity was then used as a more specific method for the measurement of the antioxidant activity. Both extracts presented a concentration-dependent SOD-like activity; however, the EC_50_ for the Misiones extract was lower than that exhibited by the Salta extract (363 vs. 537 µg/ml, Fig. 4a). In Fig. 4b–d the EC_50_ values for this activity in comparison with the polyphenols, flavonoids and tannins content is shown.

**Fig. 5:**
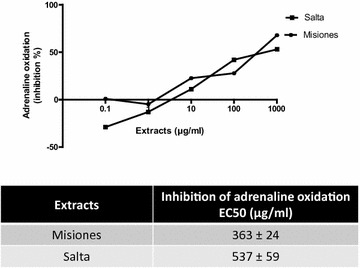
SOD like activity. **a** Percentage of inhibition of adrenaline oxidation as a function of the extract concentration. **b**–**d** Comparison between the adrenaline oxidation inhibition (SOD-like activity) and the polyphenols, flavonoids and tannins content and EC_50_ for Salta and Misiones extracts. Results represent mean ± SEM of three experiments performed in triplicate. Significant differences between extracts EC_50_ values were determined by the *t*-Student test (*p < 0.05)

To complete the antioxidant activity profile, the inhibition of lipid peroxidation of egg yolk was assayed. Results indicated that the Salta extract exerted an inhibitory effect that was higher than that of the Misiones extract, exhibiting a lower EC_50_ value (1862 μg/ml) and a maximum effect of 52% of inhibition at 2 mg/ml. On the other hand, the Misiones extract also inhibited lipid peroxidation (EC_50_: > 2000; maximum inhibition: 16.7%, Fig. 5a). It noteworthy that the Misiones extract, at 100 µg/ml, exerted a pro-oxidant action, while the Salta extract, at 100 µg/ml, caused an inhibition of lipid peroxidation of 12%. Figure 5b–d show the EC_50_ value for this activity in comparison with the polyphenols, flavonoids and tannins content.

**Fig. 6:**
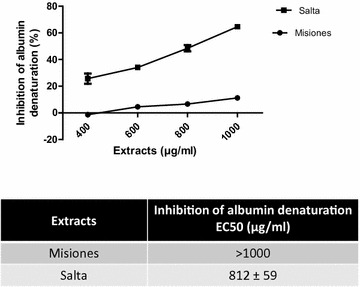
Inhibition of albumin denaturation. **a** Percentage of inhibition of albumin denaturation as a function of the extract concentration. **b**–**d** Comparison between the albumin denaturation inhibitory action and the polyphenols, flavonoids and tannins content and EC_50_ for Salta and Misiones extracts. Results represent mean ± SEM of three experiments performed in triplicate. Significant differences between extracts EC_50_ values were determined by the *t*-Student test (*p < 0.05)

The in vitro anti-inflammatory activity was studied in relation with the antioxidant activity by two methods: the inhibition of albumin denaturation and the proteinase inhibitory action. As shown in Fig. 6a, both extracts inhibited the heat-induced albumin denaturation; however, the Salta extract presented a maximum inhibition of 64.4% at 1000 μg/ml, as compared to the Misiones extract with which the maximum inhibition achieved was 11.2%, at the same concentration. In addition, the Salta extract exhibited a lower EC_50_. In Fig. 6b–d, the EC_50_ values are shown in comparison with each compound content.

**Fig. 7:**
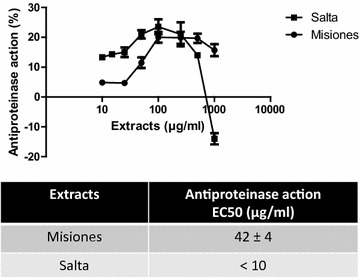
Proteinase inhibitory action. **a** Percentage of trypsin inhibition as a function of the extract concentration. **b**–**d** Comparison between the proteinase inhibitory action and the polyphenols, flavonoids and tannins content and EC_50_ for Salta and Misiones. Results represent mean ± SEM of three experiments performed in triplicate. Significant differences between extracts EC_50_ were determined by the *t*-Student test (**p < 0.01)

Finally, the trypsin inhibitory capacity was higher for the Salta extract than for the Misiones extract, showing a lower EC_50_ value (Fig. 7a). In Fig. 7b–d, the relationship between this activity and the compounds content is shown.

Finally, the effect on the nitrosative stress was studied on macrophages stimulated or not with LPS, which is an inductor of NO. The percentage of NO produced and the effect on cell proliferation were studied.

**Fig. 8:**
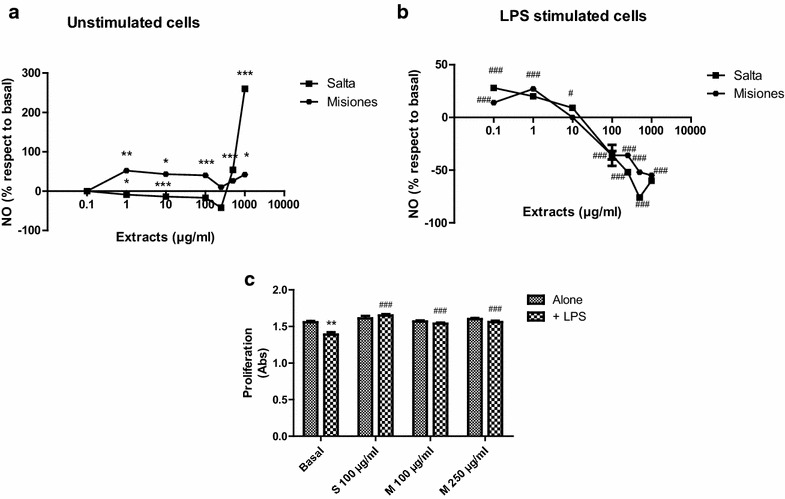
Effect of the extracts on RAW264.7 macrophages. Cells were incubated for 24 h in the presence of the extracts alone or with LPS. **a** Effect of different concentrations of the extracts on NO production without LPS stimulation. **b** Effect of different concentrations of the extracts on NO production in the presence of LPS. **c** Effect of the extracts on macrophages proliferation in the absence or the presence of LPS. Results represent mean ± SEM of three experiments performed in triplicate. Statistical differences from the control group were determined by ANOVA + Dunnett’s test (*p < 0.05; **p < 0.01; ***p < 0.001 vs. control group; ^#^p < 0.05; ^###^p < 0.001 vs. LPS group)

Figure 8a shows that in unstimulated macrophages, low concentrations of the Salta extract decreased the NO production, while higher concentrations increased the production of this mediator. On the other hand, the Misiones extract increased the NO production at all the concentrations assayed. LPS increased the NO release by macrophages (data not shown). Neither the Salta nor the Misiones extract modified cell proliferation at any concentration, but LPS was capable of decreasing cells proliferation. Under LPS stimulation, the Salta and Misiones extracts presented a biphasic effect, increasing the NO production at low concentrations and decreasing it at higher ones. The effect exerted by the Salta extract at high concentrations was higher than that of the Misiones extract (Fig. 8b). Moreover, the Salta extract, at 100 µg/ml, and the Misiones extract, at 100 and at 250 µg/ml were capable of reversing the antiproliferative effect exerted by LPS.

## Discussion

In this work the polyphenol-related antioxidant activity of two extracts of *Urera aurantiaca* from different geographical zones form Argentina was demonstrated.

DPPH.is a stable free radical with a maximum absorbance at 517 nm. This free radical can readily be scavenged by an antioxidant to be transformed into 1,1-diphenyl-2-picrylhydrazine. The degree of bleaching indicates the scavenging power of the extract. In this assay, the Salta extract presented higher scavenging activity and potency than the Misiones extract.

The extracts also proved to have reducing power. This effect was assessed by direct electron donation in the reduction of [Fe(CN)_6_]^3−^ to [Fe(CN)_6_]^4−^ [29]. The product was visualized by the appearance of the intense Prussian blue coloured complex, whose absorbance was measured at 700 nm. The Salta extract had a higher capacity (lower EC_50_) than the Misiones extract in reducing the complex Fe^3+^/ferricyanide, reaching the highest inhibitory activity. The antioxidant effect exerted by reductants is based on the interruption of free radical chain reaction by donation of a hydrogen atom. Reductants also react with certain precursors of peroxide, thus preventing peroxide formation and lipid peroxidation. Our results indicate that the marked reducing power and DPPH scavenger activities exerted by the Salta extract seem to be due to the presence of polyphenols, such as flavonoids, whose content was directly proportional to such biological activity. The reducing power of these compounds is achieved through electron donation to free radicals to convert them into more stable products thus terminating the free radical chain reaction. The scavenging activity and the reducing power are known to be related to the nature of the phenolic compounds, which contribute to their electron transfer/hydrogen donating ability [30]. Thus, flavonoids generally have higher antioxidant activity because of the double bonds present in ring C, and their antioxidant ability depends on their hydroxyl group arrangement [31]. On the contrary, tannins did not appear not to be related to these effects since the Salta extract presented a higher potency than the Misiones extract and a lower tannins content.

The Salta extract showed scavenger activity and reducing power that were directly proportional to the inhibition on lipid peroxidation. Moreover, the inhibition of lipid peroxidation correlated positively with the presence of flavonoids and not with the presence of tannins. The inhibition of lipid peroxidation might be due to the hydrogen-donating capacity of phenolic derivatives and subsequent radical stabilization.

On the other hand, the SOD-like activity was higher in the Misiones extract, and such activity correlated with a higher tannin content, as compared to the Salta extract. The SOD-like activity exerted by the extract is highly desirable, since such activity would contribute to the elimination of the superoxide radical, which causes tissue injury, including carcinogenesis, inflammation, and aging. Many clinical trials have been carried out to assess the efficacy of SOD to treat several diseases caused by the generation of high levels of superoxide. However, the treatment must be administered by the parenteral route, since SOD is inactivated by digestive enzymes and gastric juices. An alternative approach is to find low molecular weight compounds that mimic SOD or which can enhance its enzymatic activity. Such mimicry was achieved by the Misiones extract.

It is known that oxidative stress induces inflammation and vice versa. In this regard, the denaturation of proteins is a well-documented cause of inflammation, and ROS are known to stimulate this process. Salicylic acid has been demonstrated to have a dose-dependent ability to induce thermal protein denaturation by ROS generation. Proteolytic enzymes, such as trypsin, which are essential modulators of the inflammatory response [32], are also activated by ROS. To complete the study of antioxidant activity, the effect of the extracts was assayed on albumin denaturation and on tryptic casein hydrolysis. As expected, the Salta extract exerted a higher antioxidant activity, as evidenced by the lower EC_50_ on both models. These effects were related to the polyphenol content, especially flavonoids. The EC_50_ of the extracts for the DPPH scavenger, reducing power and SOD like activities were lower or equal (depending on the extract) than the EC_50_ for lipid peroxidation, inhibition of albumin denaturation and protease inhibition. These results may suggest that an antioxidant activity is required to induce the anti-inflammatory actions.

Nitric oxide is a diffusible free radical that plays many roles as an effector molecule in diverse biological systems, including neuronal messenger, vasodilatation and antimicrobial and antitumor activities [33]. It is a potent pleiotropic inhibitor of physiological processes such as smooth muscle relaxation, neuronal signaling, inhibition of platelet aggregation, and regulation of cell mediated toxicity. It is known that NO induces cell death by apoptosis. Macrophages can produce high amounts of NO upon activation by cytokines and LPS. After stimulation with LPS, macrophages secrete several pro-inflammatory products such as TNF-α, interleukins, and NO. Many transcriptor factor families, including NF-kB, activator protein 1 (AP-1), among others, are considered critical regulators of gene expression in the setting of the inflammatory process. Although these factors play an essential beneficial role in physiological processes, the deregulation of NF-kB activity and the sustained production of pro-inflammatory cytokines and NO are involved in the pathogenesis of several diseases [34].

In LPS-stimulated macrophages, both extracts were capable of decreasing the production of NO only at high concentrations, thus blunting the effect of LPS. Moreover, the extracts were able to revert the antiproliferative effect of LPS. All these effects suggested an antioxidant and anti-inflammatory activity at high concentrations. Low concentrations of the extracts had the opposite effect in NO production, increasing it without modification of cell proliferation. This protective effect of the extracts could be related to their antioxidant activity and to the presence of polyphenols. Many studies have shown that many flavonoids and related polyphenols contribute significantly to the anti-inflammatory activities exerted by many plants [35, 36].

In relation to NO production, it can be suggested that the extracts also exert an immunomodulatory activity on cells without LPS activation, demonstrating a pro-oxidant activity at low and high concentrations. The Salta extract appeared to be more effective in reducing the production of NO.

The differences observed in the antioxidant activities of the extracts could be related to a quantitative rather to a qualitative difference in the polyphenols content. These quantitative differences observed between both extracts could be a consequence of genotypic, developmental and environmental factors. It is known that specimens of same plant species growing under different environmental conditions show significant differences in the production and accumulation the primary and secondary metabolites [37]. Influenced by environmental factors, secondary metabolites act as a chemical interface between the plant and its environment. The chemical interaction between plants and their environment is mediated mainly by the biosynthesis of secondary metabolites, which exert their biological roles as a plastic adaptive response to their environment.

**Fig. 9:**
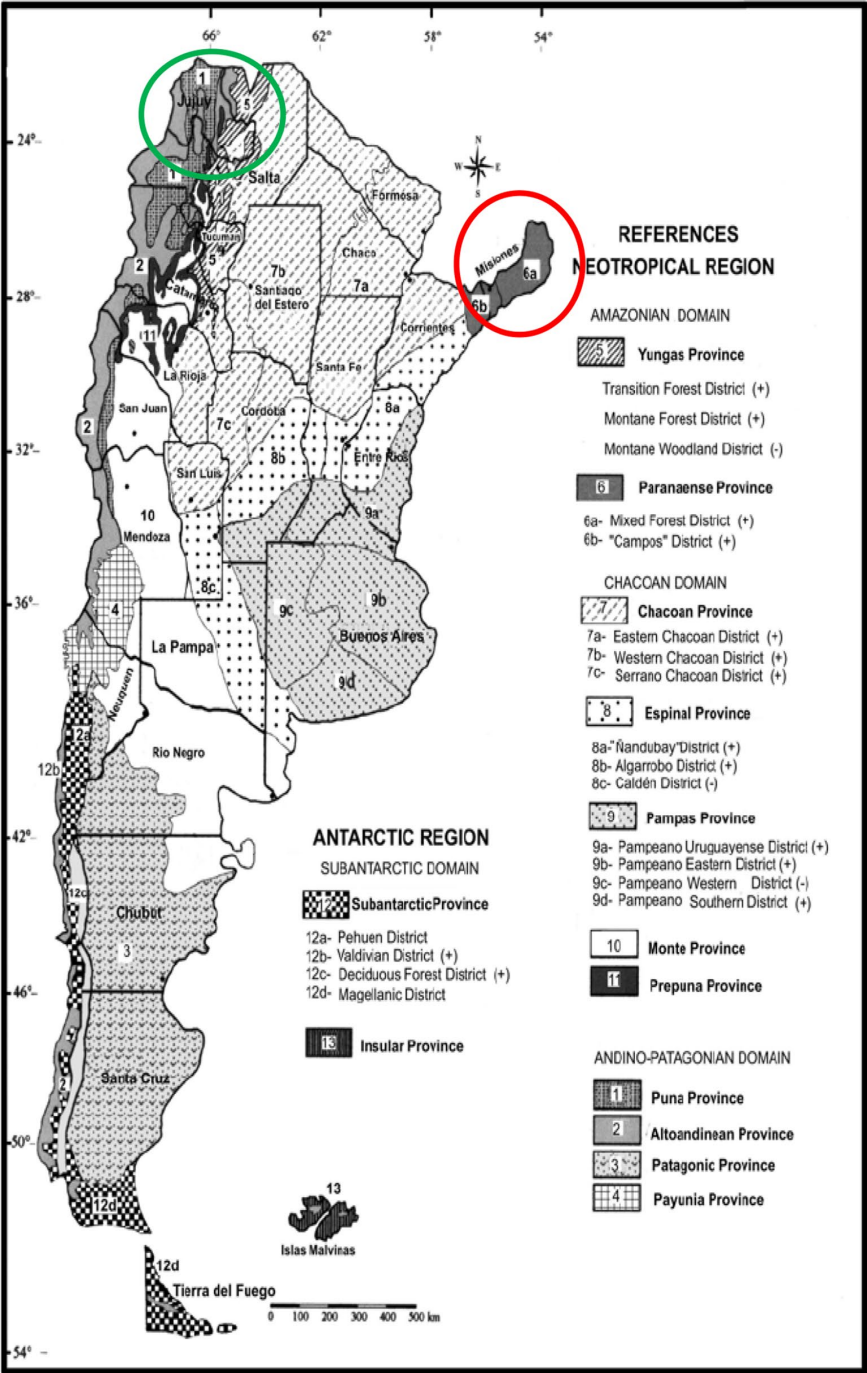
Phytogeographic Argentinean provinces and *Urera aurantiaca* distribution. Paranaense phytogeographic Province and Yungas phytogeographic province are marked in red and green, respectively

The Salta specimen was collected in the Oran zone (phytogeographic province of the Yungas) and the Misiones specimen was collected in Eldorado (Paranaense phytogeographic province) [38] (Fig. 9). These phytogeographic zones differ in climate, temperature and precipitations, factors which can influence the production of primary and secondary metabolites. In this sense, the Paranaense province is a moist forest with subtropical climate, whereas the Yungas is a rain mountain forest which constitutes a subtropical complex biome with extreme temperatures that range from 50 °C in summer to 10 °C in winter. The climate is wet and hot, with summer precipitations and winter frost. In fact, it is a very rainy and foggy zone. These differences between the biomes could explain the differences observed in the specimens.

## Conclusion

The present study demonstrated the in vitro antioxidant and anti-inflammatory activities of two methanol extracts of *U. aurantiaca*. The presence of flavonoids and other polyphenols in the extracts may be responsible for the biological activity observed. The differences found between the extracts effectiveness are probably related to ambient conditions: temperature, rain, soil composition and ecosystem.

## Additional file

**Additional file 1.** Minimum standards of reporting checklist.

## Abbreviations

DPPH: 2,2-diphenyl-1-picrylhydrazyl
LPS: lipopolysaccharide
MTT: 3-(4,5-dimethylthiazol-2-yl)-2,5-diphenyltetrazolium bromide
NO: nitric oxide
RNS: reactive nitrogen species
ROS: reactive oxygen species
SOD: superoxide dismutase
